# Identification and Antifungal Mechanism of a Novel Actinobacterium *Streptomyces huiliensis* sp. nov. Against *Fusarium oxysporum* f. sp. *cubense* Tropical Race 4 of Banana

**DOI:** 10.3389/fmicb.2021.722661

**Published:** 2021-11-04

**Authors:** Dengfeng Qi, Liangping Zou, Dengbo Zhou, Miaoyi Zhang, Yongzan Wei, Lu Zhang, Jianghui Xie, Wei Wang

**Affiliations:** ^1^Key Laboratory of Biology and Genetic Resources of Tropical Crops, Ministry of Agriculture, Institute of Tropical Bioscience and Biotechnology, Chinese Academy of Tropical Agricultural Sciences, Haikou, China; ^2^College of Life Science, Hainan Normal University, Haikou, China

**Keywords:** *Streptomyces*, novel species, identification, banana Fusarium wilt, antifungal activities, genome sequencing

## Abstract

Banana is an important fruit crop. *Fusarium* wilt caused by *Fusarium oxysporum* f. sp. *cubense* tropical race 4 (Foc TR4) seriously threatens the global banana industry. It is difficult to control the disease spread using chemical measures. In addition, commercial resistant cultivars are also lacking. Biological control is considered as a promising strategy using antagonistic microbes. Actinomycetes, especially *Streptomyces*, are potential sources of producing novel bioactive secondary metabolites. Here, strain SCA2-4^*T*^ with strong antifungal activity against Foc TR4 was isolated from the rhizospheric soil of *Opuntia stricta* in a dry hot valley. The morphological, physiological and chemotaxonomic characteristics of the strain were consistent with the genus *Streptomyces*. Based on the homology alignment and phylogenetic trees of 16S *rRNA* gene, the taxonomic status of strain SCA2-4^*T*^ exhibited a paradoxical result and low bootstrap value using different algorithms in the MEGA software. It prompted us to further discriminate this strain from the closely related species by the multilocus sequence analysis (MLSA) using five house-keeping gene alleles (*atpD*, *gyrB*, *recA*, *rpoB*, and *trpB*). The MLSA trees calculated by three algorithms demonstrated that strain SCA2-4^*T*^ formed a distinct clade with *Streptomyces mobaraensis* NBRC 13819^*T*^. The MLSA distance was above 0.007 of the species cut-off. Average nucleotide identity (ANI) values between strain SCA2-4^*T*^ genome and two standard strain genomes were below 95-96% of the novel species threshold. Strain SCA2-4^*T*^ was assigned to a novel species of the genus *Streptomyces* and named as *Streptomyces huiliensis* sp. nov. The sequenced complete genome of SCA2-4^*T*^ encoded 51 putative biosynthetic gene clusters of secondary metabolites. Genome alignment revealed that ten gene clusters were involved in the biosynthesis of antimicrobial metabolites. It was supported that strain SCA2-4^*T*^ showed strong antifungal activities against the pathogens of banana fungal diseases. Extracts abstracted from the culture filtrate of strain SCA2-4^*T*^ seriously destroyed cell structure of Foc TR4 and inhibited mycelial growth and spore germination. These results implied that strain SCA2-4^*T*^ could be a promising candidate for biological control of banana *Fusarium* wilt.

## Introduction

Banana (*Musa* spp.) is one of the most popular fruits in the tropical and sub-tropical regions of the world. It is considered to be the fourth most important crop following rice, wheat and corn in the developing countries (Mohandas and Ravishankar, 2016). Banana *Fusarium* wilt seriously threatened the global banana industry. The disease was caused by the soil-borne fungi, containing four physiological races (abbreviated as Foc 1–Foc 4) based on their host specificity. Especially, tropical race 4 (Foc TR4) can infect almost all banana cultivars (Ploetz, 2015). They can survive in the soil as chlamydospores for more than 20 years (Ploetz, 2015). It is difficult to control the disease spread using chemical measures. In addition, commercial resistant cultivars are also lacking. Biological control is considered as a promising strategy using antagonistic microbes.

Actinobacteria are rich in bioactive secondary metabolites exhibiting antimicrobial, antitumor and/or antiviral activities (Watve et al., 2001; Guo et al., 2008). These microbes are widely distributed in diverse ecosystems such as soil, air and salt water. More than 50% of the known antibiotics come from Actinobacteria. The genus *Streptomyces* is the largest genus of Actinobacteria. Over 1000 species of *Streptomyces* are identified^1^. Notably, 75% of antibiotics are produced by *Streptomyces* species (Bérdy, 2005). *Streptomyces* have been used as biocontrol agents against soil-borne diseases such as Foc TR4. *Streptomyces* g10 isolated from a coastal mangrove exhibited a strong antagonistic activity against banana *Fusarium* wilt (Getha et al., 2005). Endophytic *Streptomyce*s sp. S76 and *Streptomyce*s sp. S96 showed antifungal activity against Foc TR4 (Cao et al., 2004, 2005). We previously reported *Streptomyces* sp. CB-75, SCA3-4, and JBS5-6 isolated from rhizosphere soil strongly inhibited mycelial growth of Foc TR4 and spore germination (Chen et al., 2018; Qi et al., 2019; Jing et al., 2020). However, *Streptomyces* species are still the most potential candidates to produce novel types of secondary metabolites (Bérdy, 2005). Based on their genome information, more than 90% of *Streptomyces* bioactive metabolites are waiting to be discovered (Watve et al., 2001; Guo et al., 2008). Therefore, the isolation and identification of *Streptomyces* remains a perennial attention.

In our present study, strain SCA2-4^*T*^ was isolated from a rhizosphere soil sample of *Opuntia stricta* in a dry hot valley. The alignment of 16S *rRNA* gene and multilocus sequence analysis (MLSA) indicated that the strain had the closest taxonomic relationship with *Streptomyces orinoci* NBRC 13466^*T*^ and *Streptomyces mobaraensis* NBRC 13819^*T*^. The MLSA distance and average nucleotide identity (ANI) demonstrated that the strain belonged to a novel *Streptomyces* species, named with *Streptomyces huiliensis* sp. nov. Extracts of strain SCA2-4^*T*^ exhibited a strong antifungal activity against Foc TR4. Bioinformatics analysis showed that the strain genome contained a number of genes coding antifungal secondary metabolites, suggesting that it will have an application potential in biocontrol for phytopathogenic fungi.

## Materials and Methods

### Isolation of Actinobacteria

Soil sample was collected from rhizosphere of *Opuntia stricta* in a dry hot valley of the Huili County, Sichuan Province, China in 2014. One gram of soil sample was homogenized in 9 mL of sterile water and heated at 55°C for 20 min. The homogenate was diluted by a serial dilution method and spread on a starch casein agar (SCA) medium (Kuster and Williams, 1964) (10 g of soluble starch, 0.3 g of casein, 2.0 g of KNO_3_, 2.0 g of NaCl, 2.0 g of K_2_HPO_4_, 0.05 g of MgSO_4_⋅7H_2_O, 0.02 g of CaCO_3_, 0.01 g of FeSO_4_⋅H_2_O, and 18 g of agar in 1 L of sterile water, pH 7.0–7.4) supplemented with 50 mg/L of K_2_Cr_2_O_7_ and 50 mg/L of nystatin. The plates were incubated at 28°C for 7 days. Colonies with different morphological characteristics were selected and purified on the YE agar medium (4 g of yeast extract, 10 g of malt extract, 4 g of glucose and 20 g of agar in 1 L of distilled water, pH 7.3). The purified isolate was stocked in 20% (v/v) of glycerol at −80°C.

### Antifungal Activity of Actinobacteria

Antagonistic activities of the purified actinobacteria against Foc TR4 (ATCC 76255) were screened on potato dextrose agar (PDA) plates using a conventional spot inoculation method (Qi et al., 2019). A hyphal block (5-mm diameter) of Foc TR4 was inoculated on the center of PDA plate. Four blocks (5-mm diameter) of the isolates were placed on four symmetrical points of PDA plate. Three replicates were set for each experiment. The plate inoculated only with pathogen was used as a control. Antifungal activity was detected until pathogenic mycelium covered the whole plate in control group at 28°C. The growth diameters of Foc TR4 were measured using a cross method (Qi et al., 2019). The inhibitory zone and the percentage of fungal growth inhibition were calculated using the following formula:


The inhibitory percentage of growth=[(C-T)/C]×100%

where C and T represented average growth diameters of tested pathogen in the control and treated plates, respectively (Aghighi et al., 2004).

Based on its strong antifungal activity against Foc TR4, strain SCA2-4^*T*^ was selected as a target strain to determine antifungal activity against other fungi including *F. oxysporum* f. sp. *cubense* Race 1 (Foc 1; ACCC 31271), *Curvularia fallax* (ATCC 38579), and *Curvularia lunata* (ACCC 36365). These fungi were kindly provided by the Institute of Environment and Plant Protection, China Academy of Tropical Agricultural Sciences, Haikou, China.

### Phylogenetic and Genomic Analyses of Strain SCA2-4^*T*^

Extraction of genomic DNA was performed as described by Ahmad et al. (2017). The whole genome of strain SCA2-4^*T*^ was sequenced and assembled by the Shanghai Majorbio Bio-pharm Technology Co., Ltd., The complete 16S *rRNA* gene selected from its genome sequence was aligned with the EzTaxon server (Yoon et al., 2017a) and the NCBI database (Altschul et al., 1990). The phylogenetic trees were generated by the three algorithms including the Neighbor-Joining method, the Maximum-Likelihood method and the Maximum-Parsimony method in MEGA 7.0 (Kumar et al., 2016). The evolutionary distance was calculated by the Kimura’s two-parameter model (Kimura, 1980). The confidence level of phylogenetic tree was investigated using the bootstrap analysis on 1000 replicates (Felsenstein, 1985). In addition, five standard housekeeping genes including *atpD* (ATP synthase, beta subunit), *gyrB* (DNA gyrase B subunit), *recA* (recombinase A), *rpoB* (RNA polymerase, beta subunit), and *trpB* (tryptophan synthetase, beta subunit) were chosen from strain SCA2-4 ^*T*^ genome to perform a multilocus sequence analysis (MLSA) (Brady et al., 2008). The length of 9399 bp gene sequences including concatenated *atpD*, *gyrB*, *recA*, *rpoB* and *trpB* were compared with the five housekeeping genes of 16 related standard strains from GenBank (Supplementary Table 1). The MLSA phylogenetic trees were constructed using the Neighbor-Joining method (Saitou and Nei, 1987) the Maximum-Likelihood (Felsenstein, 1981) and the Maximum-Parsimony (Fitch, 1971). Average nucleotide identity (ANI) between genomes was calculated using the online OrthoANI (Yoon et al., 2017b). The G + C content of strain SCA2-4^*T*^ was estimated in the light of sequenced complete genome. The genome sequences of standard strains were downloaded from the public genome database of EzBioCloud^2^.

### Morphological Characteristics

The morphological profiles of strain SCA2-4^*T*^ were observed using a scanning electron microscopy (ZEISS, Germany) after inoculation on YE agar medium at 28°C for 21 days. The sample was prepared as described by Kumar et al. (2014). The growth characteristics of strain SCA2-4^*T*^ were detected on six standard ISP media, containing yeast extract-malt extract agar (ISP2 or YE), oatmeal agar (ISP3), inorganic salts-starch agar (ISP4), glycerol-asparagine agar (ISP5), peptone yeast-iron agar (ISP6), and tyrosine agar (ISP7) (Shirling and Gottlieb, 1966). The formation of melanoid pigments was observed on ISP6 and ISP7 at 28°C for 14 days. Colors of substrate and aerial mycelia as well as diffusible pigments were judged by comparing with the ISCC-NBS color charts (Kelly, 1958). The tolerance of NaCl (0–9%, at intervals of 1%, w/v) and temperature (16–46°C, at intervals of 1°C) for growth was examined on the YE plates. The effect of pH on growth was evaluated in the NB broth (1.0 g of yeast powder, 0.8 g of beef extract, 2.0 g of casein, and 10.0 g of glycerol) with pH 4–10 (at intervals of 1) at 28°C for 14 days. Physiological and biochemical characteristics were determined according to the methods of Williams et al. (1983).

### Chemotaxonomic Characteristics

The biomass of strain SCA2-4^*T*^ was collected by centrifugation at 8000 rpm for 20 min after growing in a shaken flask of the TSB broth (15.0 g of tryptone, 5.0 g of soytone, 30.0 g of sodium chloride, 1 L of sterile water, pH 7.1–7.5) at 25°C for 5 d. Respiration quinone was extracted from the lyophilized mycelia (0.5 g) by a reverse-phase partition high-performance liquid chromatography (HPLC) (Sasser, 1990). The cellular fatty acids were analyzed according to the standard Microbial Identification System (Sherlock Version 6.1, MIDI database) by a gas chromatography (56890N, Agilent Technologies) (Komagata and Suzuki, 1987). The polar lipids were extracted from dry mycelia and individually separated by a two-dimensional thin-layer chromatography (TLC) on silica gel plates (Art 5554, DC-Alufolien Kieselgel 60F 254, Merck, Germany) (Minnikin et al., 1979).

### Bioinformatic Analysis of Strain SCA2-4^*T*^

The complete genome of strain SCA2-4^*T*^ was sequenced using a paired-end sequencing method in the Illumina Hiseq × Ten platform (Illumina, San Diego, CA, United States) by the Shanghai Majorbio Bio-pharm Technology Co., Ltd., (Shanghai, China). Data were analyzed on the free online platform of the Majorbio Cloud Platform^3^. Clean sequencing data were optimized using the GapCloser v1.12 (Luo et al., 2012) and were subsequently assembled using the SOAPdenovo v2.04. The protein-coding genes were predicted by the Glimmer v3.02 (Delcher et al., 2007^4^). Gene functions were annotated using six databases (NR, Swiss-Prot, Pfam, EggNOG, GO, and KEGG). The biosynthetic gene clusters of secondary metabolites were predicted using the online antiSMASH v 6.0.1 software (Kai et al., 2017^5^).

### Recovering Extracts From the Culture Filtrate of Strain SCA2-4^*T*^

In order to determine whether secondary metabolites of strain SCA2-4^*T*^ can inhibit growth of Foc TR4 or not, it was first fermented in the broth medium (15 g of corn flour, 10 g of glucose, 0.5 g of K_2_HPO_4_, 0.5 g of NaCl, 0.5 g of MgSO_4_, 3 g of beef extract, 10 g of yeast extract, 10 g of soluble starch, 2 g of CaCO_3_, pH 7.2–7.4) at 150 rpm and 28°C for 7 days. The secondary metabolites in fermentation broth were extracted with ethyl acetate (v/v = 1:1). After mycelia were removed with the Whatman No.1 filter, secondary metabolites in organic solvent were collected using a separating funnel and evaporated using a rotary vacuum evaporator (EYELA, N-1300, Japan). The extracts were dissolved in 10% of dimethyl sulfoxide (DMSO) with 20 mg/mL of a final concentration.

### Analysis of Minimum Inhibitory Concentration and Antifungal Activity of Extracts

Minimum inhibitory concentration (MIC) of extracts against Foc TR4 was analyzed using a 96-well plate (Nunc MicroWell, Roskilde, Denmark) (Wang et al., 2013). The stock solution was sterilized through a 0.22-μm sterile filter (Millipore, Bedford, MA, United States) and diluted to a concentration range of 100–0.781 μg/mL by a two-fold serial dilution method (Qi et al., 2019). Each well contained 80 μL of the Roswell Park Memorial Institute (RPMI) mycological media, one hundred microliters of fungal suspension at 1.0 × 10 ^5^ CFU (colony-forming units)/mL and 20 μL of extract solution. Twenty microliters of 10% DMSO replacing extract solution was used as a negative control. The same volume of cycloheximide (50–0.391 μg/mL) or nystatin (50–0.391 μg/mL) replacing extract solution was used as a positive control. The 96-well plate was covered with a plastic membrane and cultured at 150 rpm for 24 h at 28°C. MIC value was determined as our description (Qi et al., 2019). Antifungal activity of extracts against Foc TR4 was assessed using an agar-well diffusion method (Tepe et al., 2005). 100 μL of extracts (16 × MIC) was added to each well. An equal amount of DMSO (10%, v/v) was used as a control. A 5 mm-diameter hyphal disk was placed on the plate center. The growth diameter of fungi was measured after cultured 28°C for 7 days. All experiments were repeated in triplicate. The growth inhibition percentage was calculated according to the above method.

### Effect of Extracts on Cell Structure of Foc TR4

Effect of strain SCA2-4^*T*^ extracts on cell structure of Foc TR4 was observed by a transmission electron microscope (TEM, HT7700, Hitachi, Japan) (Nogueira et al., 2010). Foc TR4 mycelia exposed to extracts were fixed with glutaraldehyde (2.5%, v/v) for 3 h and washed three times with PBS (0.1 mol/L, pH 7.0). Foc TR4 mycelia treated with 10% (v/v) of DMSO were used as a control. Mycelial samples were postfixed with 1% (w/v) of osmium tetroxide in PBS (0.1 mol/L, pH 7.3) for 3 h at room temperature and rinsed three times with same buffer. Thereafter, samples were dehydrated in a graded ethanol solution (70, 80, 90, 95, and 100%) for 20 min, respectively. Dehydrated specimens were then embedded in the Epon 812 resin at 37°C for 12 h, 45°C for 12 h, and 60°C for 24 h, respectively. The samples were cut into ultrathin sections (approximately 50 nm in thickness) using an ultramicrotome (EM UC6, Leica, Germany). After stained with saturated uranyl acetate and lead citrate, the sections were observed using TEM.

### Effect of Extracts on Spore Germination of Foc TR4

Effect of strain SCA2-4^*T*^ extracts on spore germination of Foc TR4 was examined by an optical microscope (Axio Scope A1, Carl ZEISS, Germany) (Chen et al., 2018). Foc TR4 spores were collected using a sterile L-shaped spreader and adjusted to a concentration of 1.0 × 10^5^ CFU/mL with sterile distilled water. Ten microliters of extracts and an equal volume of spore suspension were mixed in a cavity glass slide at 28°C for 20 h. Three replications were set for each experiment. One hundred spores in per slide were observed for germination. The germination rate and the inhibition rate of spore germination were calculated according to the following formula: The germination rate = (A or B)/100 × 100% and the inhibition rate of spore germination = (A−B)/A × 100%. A and B were the number of germinated spores in the control and treatment groups, respectively.

## Results and Discussion

### Isolation and Antifungal Activity Assay of Actinobacteria

**FIGURE 1:**
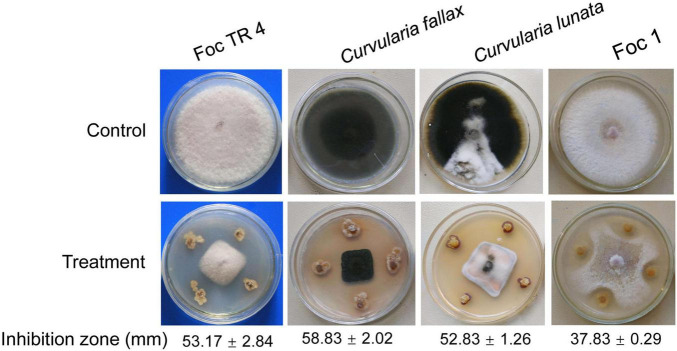
Antifungal activity of strain SCA2-4^*T*^ against four banana fungal pathogens. Foc TR4: *Fusarium oxysporum* f. sp. *cubense* tropical race 4; Foc 1: *Fusarium oxysporum* f. sp. *cubense* 1.

Forty-eight actinobacteria were isolated using the SCA medium from soil sample of the dry hot valley. Antagonistic activities against Foc TR4 were screened using a conventional spot inoculation method. Fourteen strains exhibited antifungal activities against Foc TR4. Strain SCA2-4^*T*^ had a high antifungal activity and was selected for the following study. We further assessed its antifungal activities against other phytopathogenic fungi such as Foc 1, *C. fallax* and *C. lunata* (Figure 1). Compared with the growth diameter (85.00 mm ± 0.00) in the control group, the inhibitory zones of strain SCA2-4^*T*^ against Foc TR4, Foc 1, *C. fallax*, and *C. lunata* were 53.17 mm ± 2.84, 37.83 mm ± 0.29, 58.83 mm ± 2.02, and 52.83 mm ± 1.26, respectively. The percentages of mycelial inhibition were 62.55, 44.51, 69.21, and 62.15%, respectively.

### Phylogenetic and Genomic Analyses of Strain SCA2-4^*T*^

**FIGURE 2:**
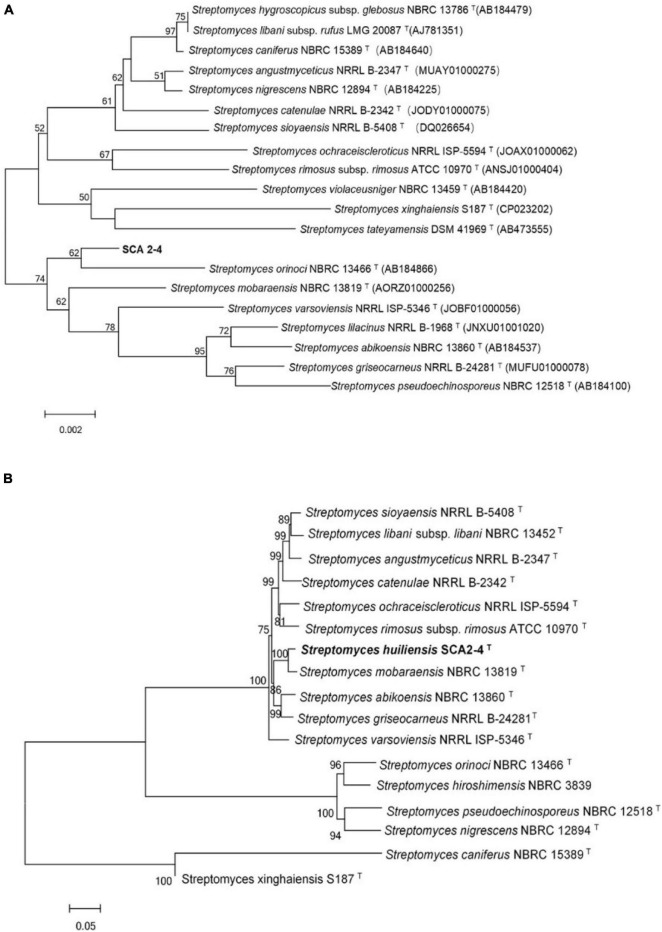
Phylogenetic trees of strain SCA2-4^*T*^ based on 16S *rRNA* and house-keeping genes. Bootstrap percentages (based on 1000 replications) were shown at branching points. Bar, 0.002 or 0.05 substitutions per nucleotide position. **(A)** Construction of phylogenetic tree based on the complete 16S *rRNA* sequences using the Neighbor-Joining method. Accession numbers of the selected genes were listed in brackets. **(B)** Construction of phylogenetic tree based on concatenated five house-keeping genes (*atpD*, *gyrB*, *recA*, *rpoB*, and *trpB*) using the Neighbor-Joining method. Strain names and accession numbers were provided in Supplementary Table 1.

Because 16S *rRNA* had a high resolving ability for relatedness between different organisms on the genus level (Stackebrandt and Goebel, 1994), these sequences were first used to identify the genus of strain SCA2-4^*T*^. A complete gene sequence of 16S *rRNA* (1523 bp, Genbank accession number MW547058) was selected from the sequenced genome of strain SCA2-4^*T*^. By aligned with the different databases, the strain belonged to a member of the genus *Streptomyces*. The 16S *rRNA* sequence of strain SCA2-4^*T*^ shared 99.17% of similarity with *Streptomyces mobaraensis* NBRC 13819^*T*^. However, the phylogenetic trees constructed by the Neighbor-joining method showed that strain SCA2-4^*T*^ fell into a subclade with *S. orinoci* NBRC 13466^*T*^ (98.89% of similarity) with 54% of bootstrap value (Figure 2A). The taxonomic status of subclade was supported by the maximum-parsimony tree with 25% of low bootstrap value, but was not supported by the maximum-likelihood tree. The low bootstrap values generated by different algorithms indicated that strain SCA2-4^*T*^ was not distinguished from closely related species using 16S *rRNA* genes. Likewise, the previous study also showed that 16S *rRNA* genes lacked sufficient resolution for closely related species due to its multiple copies and conservative feature in the single genome (Martens et al., 2008).

In order to further determine the phylogenetic status of strain SCA2-4^*T*^, the multilocus sequence analysis (MLSA) was performed using the sequences of *atpD*, *gyrB*, *recA*, *rpoB*, and *trpB* genes. The MLSA was considered as an alternative method for refining the *Streptomyces* systematics due to its efficiency of inter- and intra-species resolution and reproducibility (Rong and Huang, 2010). The Neighbor-joining tree based on MLSA demonstrated that strain SCA2-4^*T*^ formed a distinct clade with *S. mobaraensis* NBRC 13819^*T*^ with 100% of bootstrap value (Figure 2B). The taxonomic status of subclade was well supported by trees constructed by other two algorithms. These five protein-coding genes were also selected to identify *Streptomyces* (Rong et al., 2010; Huang et al., 2019). Rong and Huang (2012) found high correlation between the five-gene MLSA and the DNA-DNA hybridization in *Streptomyces*. The five-gene nucleotide sequence distance above 0.007 could be considered as an independent species. Our results showed the distance of MLSA between strain SCA2-4^*T*^ and other selected strains ranged from 0.024 to1.020 (Supplementary Table 2), suggesting that strain SCA2-4^*T*^ might be a novel species.

Although the conventional DNA-DNA hybridization was often used to clarify the species of strains, the technique was time-consuming, labor-intensive and low reproducible (Stackebrandt and Goebel, 1994; Goris et al., 2007). Average nucleotide identity (ANI) was a robust and sensitive means to compare genetic relatedness among strains by the alignment of genome sequences (Konstantinidis and Tiedje, 2005; Goris et al., 2007). However, the limited availability of strain whole-genome sequences restricted the use of ANI in taxonomic purposes. Based on the above results, we selected two standard strains (*S. orinoci* NBRC 13466^*T*^, = NRRL B-3379^*T*^, GenBank Accession No. NZ_PHNC00000000.1 and *S. mobaraensis* NBRC 13819^*T*^, = DSM 40847, GenBank Accession No. NZ_VOKX00000000.1) to further identify the species of strain SCA2-4^*T*^. We compared with strain SCA2-4^*T*^ genome (DDBJ/ENA/GenBank Accession No. JAIQLH000000000) and two standard strain genomes. ANI values were 80.44 and 90.76%, respectively, which were below the threshold value of 95–96% for distinguishing a novel species (Richter and Rossello-Mora, 2009; Chun et al., 2018). Thus, strain SCA2-4^*T*^ was identified as a novel species of the genus *Streptomyces*.

### Phenotypic Characteristics

**FIGURE 3:**
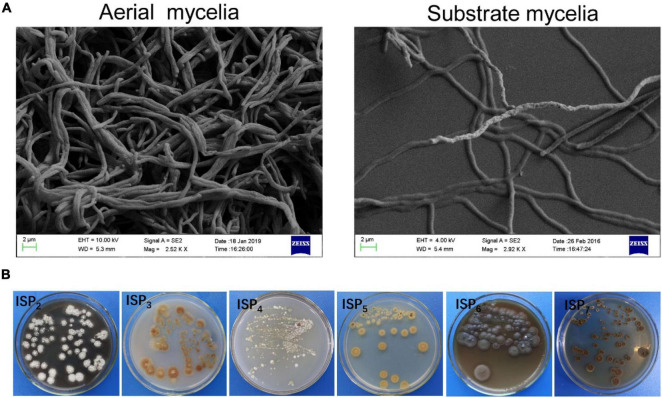
Mycelial and morphological characteristics of SCA2-4^*T*^. **(A)** Aerial and substrate mycelia were detected on the YE agar medium at 28°C for 21 days using the scanning electron microscope. **(B)** Cultural characteristics of strain 2-4^*T*^ on six ISP media.

Strain SCA2-4^*T*^ was an aerobic and Gram-positive bacterium. SEM analysis revealed that the strain produced well-developed and branched substrate and aerial mycelium that could not differentiate into spore chains (Figure 3A). It could grow well on ISP2, ISP3 and ISP6 media, followed by ISP4, ISP5 and ISP7 media. Aerial hypha demonstrated gray white, reddish brown, and earthy yellow colors on ISP2 and ISP4 media, respectively. None of aerial mycelia was produced on other two media. The hyphal substrate colors on ISP2-7 media were dark brown, light yellow, light yellow, gold, iron gray, and dark brown, respectively. Strain SCA2-4^*T*^ produced brown or light brown **s**oluble pigment on different media except for ISP3-5 (Figure 3B). These morphological and cultural characteristics were consistent with those of the genus *Streptomyces*. By contrast, two standard strains selected grew well on all ISP media. Their cultural characteristics were compared in Table 1.

**TABLE 1:** Comparison of growth characteristics between strain SCA2-4^*T*^ and closely related strains on six standard ISP media.

Medium	Aerial mycelium color			substrate mycelium color			Soluble pigment			Growth		
	1	2	3	1	2	3	1	2	3	1	2	3
Trytone-yeast extract agar (ISP2 or YE)	Gray white	White	None	Dark brown	Pastel yellow	Broom yellow	Brown	None	None	Good	Good	Good
Oatmeal agar (ISP3)	Reddish brown	White	None	Light yellow	Beige	Yellow	None	None	None	Good	Good	Good
Inorganic salts–starch agar (ISP4)	Light yellow	White	None	Light yellow	Colorless	Yellow	None	None	None	Good	Good	Good
Glycerol–asparagine agar (ISP5)	Earthy yellow	White	None	Gold	Beige	Curry	None	None	None	Good	Good	Good
Peptone–yeast extract iron agar (ISP6)	None	None	None	Iron gray	Brown red	Yellow	Brown	None	None	Good	Good	Good
Tyrosine agar (ISP7)	None	White	None	Dark brown	Brown	Yellow	Light brown	Brown	None	Good	Good	Good

*SCA2-4^*T*^; 2: *Streptomyces mobaraensis* NBRC 13819^*T*^; 3: *Streptomyces orinoci* NBRC 13466^*T*^. The data of *Streptomyces mobaraensis* NBRC 13819^*T*^ and *Streptomyces orinoci* NBRC 13466^*T*^ came from *Streptomyces mobaraensis* IPCR 16-22 | Type strain | DSM 40847, ATCC 29032, JCM 4168, KCC S-0168 | BacDiveID:16163 (dsmz.de) and Streptomyces orinoci 1882 | Type strain | DSM 40571, ATCC 23202, CBS 767.72, IFO 13466, IPV 1901, ISP 5571, JCM 4546, JCM 4807, NBRC 13466, NRRL B-3379, RIA 1427 | BacDiveID:16172 (dsmz.de).*

In addition, strain SCA2-4^*T*^ could grow at pH from 5.0 to 8.0 (optimum pH 7.0), temperature from 17–45°C (optimum 28°C) and NaCl from 0 to 6% (w/v). It could degrade Tween 80, tyrosine, gelatin and nitrate, but not resolve urea, Tween 20, starch and cellulose. Strain SCA2-4^*T*^ produced melanoid pigment, but not H_2_S. It could utilize carbon sources such as L-Arabinose, D-fructose, D-glucose, inositol, D-raffinose, L-rhamnose, and sucrose, but not Cellulose, D-mannitol and D-xylose (Table 2). In addition, strain SCA2-4^*T*^ used L-phenylalanine, ammonium sulfate, L-hydroxyproline, L (+)-cysteine, histidine, glycine, valine, and ammonium oxalate as sole nitrogen sources, but not ammonium acetate, ammonium nitrate, ammonium molybdate tetrahydrate, L-arginine and glutamate. The strain was sensitive to rifampicin, but resistant to ampicillin, chloramphenicol, streptomycin, penicillin-G, gentamicin, nystatin, tetracycline, neomycin sulfate and kanamycin sulfate (Supplementary Table 3).

**TABLE 2:** Morphological, physiological and biochemical characteristics of strain SCA2-4^*T*^ and two standard strains.

Characteristics	Strain 1	Strain 2	Strain 3
**Morphology**	Branched substrate and aerial mycelium, which could not differentiate into spore chains	Umbellate monoverticillate morphology, cylindrical spores with smooth surface	Branched vegetative mycelium, straight spore filaments, cylindrical spores with smooth surface

**Physiological**			
Temperature range for growth (?)	1745°C (optimum 28 C)	>45°C	>50°C
PH range for growth	PH5-8 (optimum pH 7.0)	N	N
NaCl tolerance for growth (%)	<6%	≤5%	≤2.5%

**Biochemical**			
Urease production	−	+	−
Tween 20	−	N	N
Tween 80	+	N	N
Degradation of cellulose	−	N	N
Melanoid pigment	+	−	+
Tyrosinase production	+	+	−
Starch hydrolysis	−	+	−
H_2_S production	−	−	+
Gelatin liquefaction	+	−	−
Nitrate reduction	+	+	−

**Growth on sole carbon sources (1.0%, w/v)**			
L-Arabinose	+	−	±
Cellulose	−	−	±
D-fructose	+	−	±
D-glucose	+	+	±
D-mannitol	−	−	±
Inositol	+	−	±
D-raffinose	+	−	±
L-rhamnose	+	−	±
Sucrose	+	−	±
D-xylose	−	−	±

*Strain 1: SCA2-4^*T*^; Strain 2: *Streptomyces mobaraensis* NBRC 13819^*T*^; Strain 3: *Streptomyces orinoci* NBRC 13466^*T*^. The data of *Streptomyces mobaraensis* NBRC 13819^*T*^ and *Streptomyces orinoci* NBRC 13466^*T*^ came from *Streptomyces mobaraensis* IPCR 16-22 | Type strain | DSM 40847, ATCC 29032, JCM 4168, KCC S-0168 | BacDiveID:16163 (dsmz.de) and Streptomyces orinoci 1882 | Type strain | DSM 40571, ATCC 23202, CBS 767.72, IFO 13466, IPV 1901, ISP 5571, JCM 4546, JCM 4807, NBRC 13466, NRRL B-3379, RIA 1427 | BacDiveID:16172 (dsmz.de). +: positive; −: negative; ±: Positive is more than negative. N: No data.*

### Chemotaxonomic Characteristics

**FIGURE 4:**
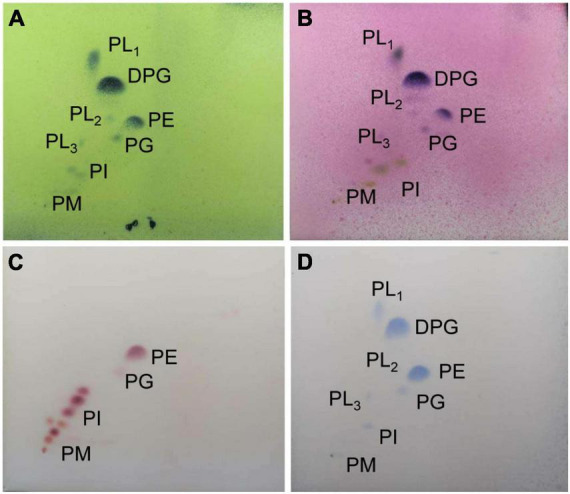
The phospholipid profile of strain SCA2-4 ^*T*^ sprayed with phosphomolybdic acid **(A)**, a-naphthol **(B)**, ninhydrin **(C)**, and phosphate **(D)**. The horizontal and vertical directions were developed using the chloroform-methanol-water (65:25:4) (v/v) and the chloroform-methanol-acetic acid-water (80:12:15:4) (v/v), respectively. DPG, diphosphatidylglycerol; PE, phosphatidylethanolamine; PL1-3, unidentified phospholipids; PG, phosphatidylglycerol; PI, Phosphatidylinositol; PIM, phosphatidylinositolmannoside.

By analysis, the predominant menaquinones of strain SCA2-4^*T*^ were MK-9(H_4_) (40.0%) and MK-9(H_6_) (44.0%). MK-_9_(H_2_) (7.0%), and MK-9(H_4_) (9.0%) were minor components. The composition of fatty acids included Antesio-C_15__:__0_ (35.01%), Iso-C_15__:__0_ (28.01%) and C_16__:__1_ω7c (11.03%) (Table 3). The major polar lipids consisted of diphosphatidylglycerol, unidentified phospholipid, and phosphatidylethanolamine. We also identified phosphatidylglycerol, two unidentified lipids, phosphatidylinositol and phosphatidylinositol mannoside (Figure 4). The chemotaxonomic characteristics of strain SCA2-4^*T*^ were consistent with those of the genus *Streptomyces*.

**TABLE 3:** Chemotaxonomic characteristics of strain SCA2-4^*T*^.

Characteristic	SCA2-4^*T*^
**Major menaquinones (%)**	
MK-9(H_2_)	7.0
MK-9(H_4_)	40.0
MK-9(H_6_)	44.0
MK-9(H_8_)	9.0

**Major fatty acids (>0.5%)**	
Saturated	
C_14__:__0_	0.74
C_15__:__0_	-
C_16__:__0_	5.1
Antesio-C_15__:__0_	35.01
Antesio-C_17__:__0_	4.27
Iso-C_13__:__0_	0.64
Iso-C_14__:__0_	1.57
Iso-C_15__:__0_	28.01
Iso-C_16__:__0_	3.01
Iso-C_17__:__0_	2.64
Unsaturated	
Iso-C_16__:__1_ H	1.67
C_16__:__1_ω7c	11.03
C_16__:__0_ 9 methyl	1.31
Antesio-C_17__:__1_ C	1.09

**Major polar lipids**	DPG, PL_1_, PE

*DPG, diphosphatidylglycerol; PL_1_, unidentified phospholipids; PE, phosphatidylethanolamine.*

### Bioinformatic Analysis

**FIGURE 5:**
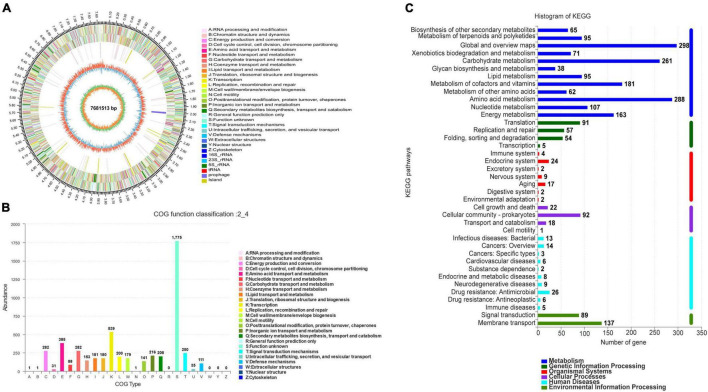
Genome information and function annotation of strain SCA2-4^*T*^. **(A)** Circular map of strain SCA2-4^*T*^ genome. From outside to center, ring 1 was the mark of genome size. Rings 2 and 3 represented CDS on forward/reverse strand. Different colors indicated the functional category of CDS. Ring 4 was tRNA and rRNA. Ring 5 showed the G + C content. The outward red part indicated that the GC content of this region was higher than the average GC content of the whole genome. The inward blue part indicated that the GC content of this region was lower than the average GC content of the whole genome, followed by G + C skew in ring 6. **(B)** The COG annotation of strain SCA2-4^*T*^ genome. **(C)** The KEGG pathway annotation of strain SCA2-4^*T*^ genome.

Strain SCA2-4^*T*^ genome was sequenced and contained 7681513 bp. The genome with 73.19% of GC content included one *rRNA* gene, sixty-nine tRNA genes and 7137 coding sequences (CDS) (Figure 5A and Table 4). By annotation, 73.41, 34.22, and 60.95% of CDS were assigned to COG, KEGG, and GO, respectively (Table 4). In the COG categories, 34.26, 17.58, and 14.28% of genes participated in metabolism, information storage and processing as well as cellular processes and signaling, respectively. Significantly, 33.88% of COG genes were unknown function (Figure 5B and Supplementary Table 4). These coding sequences were responsible for different function profiles in the KEGG pathways (Figure 5C). By analysis of antiSMASH software, fifty-one gene clusters related to the secondary metabolite biosynthesis were identified in genome sequences of strain SCA2-4^*T*^ (Supplementary Table 5). They included nine PKS I gene clusters, eight terpene gene clusters, five NRPS gene clusters, four PKS III gene clusters, three other gene clusters, two lasso peptide gene clusters, two PKS II gene clusters, two siderophore gene clusters, two otherks gene clusters, two lantipeptide gene clusters, one PKS III-siderophore gene cluster, one fused gene cluster, one arylpolyene gene cluster, one lantipeptide-lasso peptide gene cluster, one lantipeptide-NRPS gene cluster, one ladderane gene cluster, one indole gene cluster, one PKS III-terpene gene cluster, one terpene-NRPS gene cluster, one bacteriocin gene cluster, one PKS I-NRPS gene cluster and one thiopeptide gene cluster (Supplementary Table 6). The gene and chemical structures of sixteen secondary metabolites with more than 70% of sequence similarity were listed in Supplementary Figure 1.

**TABLE 4:** Genome information of strain SCA2-4^*T*^.

Items	Chromosome characteristics	% of total^a^
Chromosome size (bp)	7681513	100
DNA coding region (bp)	6448287	83.95
GC content (bp)	5621792	73.19
RNA genes^b^	70	−
Protein-coding genes	7137	100
Genes assigned to COG	5165	72.37
Genes assigned to KEGG	2442	34.22
Genes assigned to GO	4350	60.95
CRISPR repeat	114	1.6

*^*a*^The total was based on either the size of the genome in base pairs or the total number of proteins encoding genes in the annotated genome.*

*^*b*^RNA genes also included one rRNAs and 69 tRNAs.*

Genome analysis further revealed that ten gene clusters were involved in the biosynthesis of antimicrobial metabolites, including naringenin (Salehi et al., 2019), piericidin A1 (Engl et al., 2018), simocyclinone (Schimana et al., 2000), granaticin (Snipes et al., 1979), medermycin (Takano et al., 1976), cyclothiazomycin b1 (Mizuhara et al., 2011), cyclothiazomycin C (Cox et al., 2014), caboxamycin (Hohmann et al., 2009), rhizomide A-C (Wang et al., 2018), and alkylresorcinol (Dey et al., 2013). Among them, gene clusters encoding naringenin, simocyclinone, cyclothiazomycin C and alkylresorcinol showed 100% of similarity with the known genes. Thirteen gene clusters might participate in the biosynthesis of anticancer agents including naringenin, aranciamycin, AT2433 (Singh et al., 2015), rebeccamycin (Bush et al., 1987), staurosporine (Nakano and Omura, 2009), K-252a (Nakano and Omura, 2009), cladoniamide (Loosley et al., 2013), oviedomycin (Lombó et al., 2004), landomycin (Henkel et al., 1990), simocyclinone, granaticin, medermycin, and caboxamycin.

Notably, one gene cluster was responsible for in the biosynthesis of siderophore bacillibactin. Siderophores had a competition ability for ferric iron (Fe^3+^) by the receptors of the siderophore-producing strains (Sheng et al., 2020). They played an important role in enhancing plant resistance and reducing pathogen infection (Gu et al., 2020). Sheng et al. (2020) reported that *Brevibacillus brevis* GZDF3 had strong antifungal activity against *Candida albicans* by producing siderophores. The previous study showed that *Streptomyces* S96 lacked antagonistic activity against Foc TR4 by adding an excess ferric iron (Cao et al., 2005). The role of siderophore bacillibactin in antifungal activity of strain SCA2-4^*T*^ against plant pathogenic fungi including Foc TR4 needs further study. In addition, several unmatched gene clusters were also found in the genome of strain SCA2-4^*T*^, which might participate in the biosynthesis of some key secondary metabolites.

### Description of *Streptomyces huiliensis* sp. nov.

*Streptomyces huiliensis* (hui.li.en’sis. N.L. masc. adj. *huiliensis* of Huili, a county in China, referring to the place where the type strain was first isolated). The strain was Gram-positive, aerobic and non-motile. It formed branched substrate and aerial mycelia and could grow good on all of ISP agars. Aerial mycelia with gray white, reddish brown, light yellow and earthy yellow were formed on ISP2-4, but not aerial mycelia were detected on ISP6 and ISP7. Substrate mycelia were dark brown on ISP2, light yellow on ISP3-4, gold on ISP5, iron gray and dark brown on ISP6 and ISP7. Brown soluble pigments were produced on ISP2, but not on ISP3-4. Melanin was produced on ISP6 or ISP. The strain could grow at 17–45°C (optimum 28°C), pH 5–8 (optimum pH 7) and 0–6% (w/v) NaCl. It could degrade Tween 80, tyrosine, gelatin and nitrate, but not urea, Tween 20, starch and cellulose. The strain did not produce H_2_S. It utilized L-Arabinose, D-fructose, D-glucose, inositol, D-raffinose, L-rhamnose and sucrose as sole carbon sources, but not Cellulose, D-mannitol and D-xylose. It could also use L-phenylalanine, ammonium sulfate, L-hydroxyproline, L (+)-cysteine, histidine, glycine, valine and ammonium oxalate as sole nitrogen sources, but not ammonium acetate, ammonium nitrate, ammonium molybdate tetrahydrate, L-arginine and glutamate. strain SCA2-4^*T*^ was resistant to ampicillin, chloramphenicol, streptomycin, Penicillin-G, gentamicin, nystatin, tetracycline, neomycin sulfate and kanamycin sulfate, but sensitive to rifampicin.

MK-9(H_4_) (40.0%) and MK-9(H_6_) (44.0%) were found as the major menaquinones of the strain. The major fatty acids were composed of Antesio-C_15__:__0_ (35.01%), Iso-C_15__:__0_ (28.01%) and C_16__:__1_ω7c (11.03%). The major polar lipids consisted of diphosphatidylglycerol, one unidentified phospholipid, and phosphatidylethanolamine.

Strain SCA2-4^*T*^ (= GDMCC 4.215^*T*^) was isolated from the soil sample collected from rhizosphere of *O. stricta* in a dry hot valley of the Huili County, Sichuan Province, China. The 16S *rRNA* gene sequence was submitted into GenBank with accession number MW547058. This Whole Genome Shotgun project of the strain has been deposited at DDBJ/ENA/GenBank under the accession JAIQLH000000000. The version described in this paper is version JAIQLH010000000. The genome was 7681513 bp with 73.19% of GC content.

### Antifungal Activity and MIC of Extracts Against Foc TR4

**FIGURE 6:**
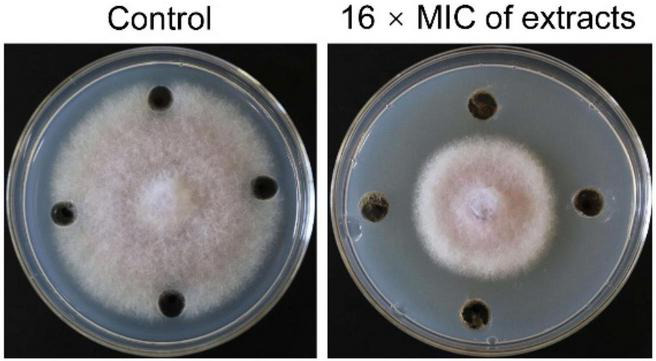
Inhibition assay of strain SCA2-4^*T*^ extracts on the mycelial growth of Foc TR4. 10% of DMSO treatment was used as control.

In order to determine whether the secondary metabolites of strain SCA2-4^*T*^ had antifungal activity or not, extracts were isolated with ethyl acetate. Minimum inhibitory concentration (MIC) of extracts ranged from 100 to 0.781 μg/mL against Foc TR4. Two standard antibiotics cycloheximide and nystatin used as positive controls. The results displayed that MIC of extracts was 6.25 μg/mL and MIC values of two standard antibiotics cycloheximide and nystatin were 1.563 and 6.25 μg/mL, respectively. The results indicated that secondary metabolites of strain 2-4^*T*^ had a strong inhibitory activity on Foc TR4 (Figure 6). Inhibition zone and mycelial inhibition rate reached 31.83 mm ± 2.36 and 42.47% (Supplementary Table 7), respectively.

### Effect of Extracts on Spore Germination and Cell Structure of Foc TR4

**FIGURE 7:**
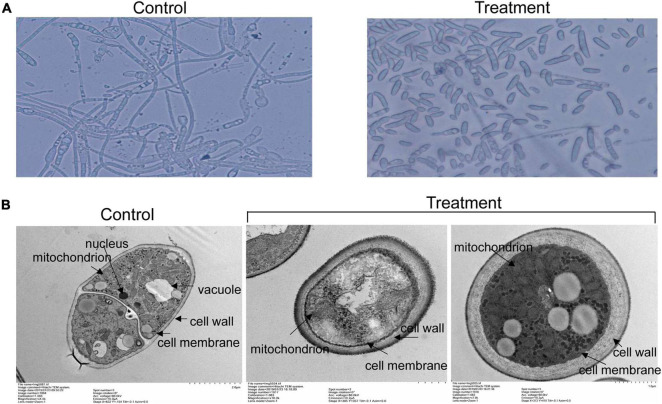
Effects of strain SCA2-4^*T*^ extracts on spore germination and cell structure of Foc TR4. 10% of DMSO treatment was used as control. **(A)** Measurement of spore germination after treated with 16 × MIC of strain SCA2-4^*T*^ extracts. **(B)** Cell structure of Foc TR4 treated with 16 × MIC of strain SCA2-4^*T*^ extracts was detected using the transmission electron microscopy.

To further analyze antifungal mechanism of strain SCA2-4^*T*^ extracts, inhibition activity of extracts on spore germination of Foc TR4 was investigated (Figure 7A). The results showed that spore germination of Foc TR4 was obviously inhibited by extracts of strain SCA2-4^*T*^. Compared with 94.33 ± 2.08% of spore germination rate in the control, the inhibition rate was 89.08% in the treatment group. In addition, cell ultrastructure of Foc TR4 was detected using TEM after treatment. By contrast, cells of Foc TR4 mycelia treated with 10% of DMSO kept an intact and discernible structure of cell membrane with the uniform cytochylema. All organelles such as mitochondria, nuclei and vacuoles had normal morphological characteristics (Figure 7B). However, fungal cell wall and cytoplasmic membrane treated with 16 × MIC of extracts became irregular and thickened. Many organelles such as nuclei and vacuoles were broken. Mitochondrial structures were disrupted, suggesting that extracts caused serious damage to cell structure of strain SCA2-4^*T*^ extracts.

## Conclusion

An actinobacterial strain SCA2-4^*T*^ was isolated from the rhizospheric soil of *O. stricta* in a dry hot valley and selected for its excellent antifungal activity against *Foc* TR4. Strain SCA2-4^*T*^ was identified as a novel species of the genus *Streptomyces* in the light of genotype and phenotype data. The strain was proposed as *Streptomyces huiliensis* sp. nov. The whole genome analysis showed that strain SCA2-4^*T*^ contained 51 putative biosynthetic gene clusters. Especially, ten gene clusters were involved in the biosynthesis of antimicrobial metabolites. The strain also exhibited high antifungal activity against pathogens of other banana fungal diseases such as Foc 1, *C. fallax*, and *C. lunata.* Extracts of strain SCA2-4^*T*^ seriously destroyed fungal cell structure of Foc TR4, inhibited the mycelial growth and spore germination. MIC was 6.25 μg/mL against Foc TR4. These results implied that this strain could be a promising candidate for biological control of banana *Fusarium* wilt.

## Data Availability Statement

The datasets presented in this study can be found in online repositories. The names of the repository/repositories and accession number(s) can be found in the article/Supplementary Material.

## Author Contributions

DQ, LiZ, DZ, LZ, JX, and WW developed the ideas and designed the experimental plans. WW and JX supervised the research and provided the fund support. DQ, LiZ, DZ, MZ, and YW performed experiments. DQ, WW, and JX provided the materials. DQ, LZ, and WW analyzed the data and prepared the manuscript. All authors contributed to the article and approved the submitted version.

## Conflict of Interest

The authors declare that the research was conducted in the absence of any commercial or financial relationships that could be construed as a potential conflict of interest.

## Publisher’s Note

All claims expressed in this article are solely those of the authors and do not necessarily represent those of their affiliated organizations, or those of the publisher, the editors and the reviewers. Any product that may be evaluated in this article, or claim that may be made by its manufacturer, is not guaranteed or endorsed by the publisher.

## Footnotes

1
http://www.bacterio.net/

2
https://www.ezbiocloud.net/search?tn=Nocardioides

3
http://www.majorbio.com/

4
http://ccb.jhu.edu/software/glimmer/index.shtml

5
http://antismash.secondarymetabolites.org/#!/start

## Supplementary Material

TABLEg S5 Genome-wide analysis of strain SCA2-4^*T*^ for gene clusters of the biosynthesis of secondary metabolites by the online antiSMASH v4.2.0 software.

Supplementary Table 1 Allele sequence accession numbers of the *Streptomyces* used for the present study.

Supplementary Table 2 MLSA distance values for selected strains in this study.

Supplementary Table 3 Nitrogen-source utilization and antibiotic sensitivity of strain SCA2-4^*T*^.

Supplementary Table 4 Functional cluster of orthologous genes (COG) classification of predicted genes in strain SCA2-4^*T*^.

Supplementary Table 5 Biosynthetic gene clusters of secondary metabolite of strain SCA2-4^*T*^ on the online antiSMASH v 6.0.1.

Supplementary Table 6 Cluster number and gene number shown in different cluster types of strain SCA2-4^*T*^.

Supplementary Table 7 Inhibitory activity and MIC of extracts of strain SCA2-4^*T*^ against Foc TR4.
