# Supplementary material for: Identification and Antifungal Mechanism of a Novel Actinobacterium Streptomyces huiliensis sp. nov. Against Fusarium oxysporum f. sp. cubense Tropical Race 4 of Banana
Source: Front Microbiol. 2021 Nov 4;12:722661. doi: 10.3389/fmicb.2021.722661 (PMC8600237; doi:10.3389/fmicb.2021.722661)
Supplement: TABLEg S5 — Genome-wide analysis of strain SCA2-4T for gene clusters of the biosynthesis of secondary metabolites by the online antiSMASH v4.2.0 software. [file Data_Sheet_1.docx]

**Identification and Antifungal Mechanism of a Novel Actinobacterium *Streptomyces huiliensis* sp. nov. against *Fusarium Oxysporum* f. sp. *Cubense* Tropical Race 4 of Banana**

Dengfeng Qi, Liangping Zou, Dengbo Zhou, Miaoyi Zhang, Yongzan Wei, Lu Zhang, Jianghui Xie, Wei Wang


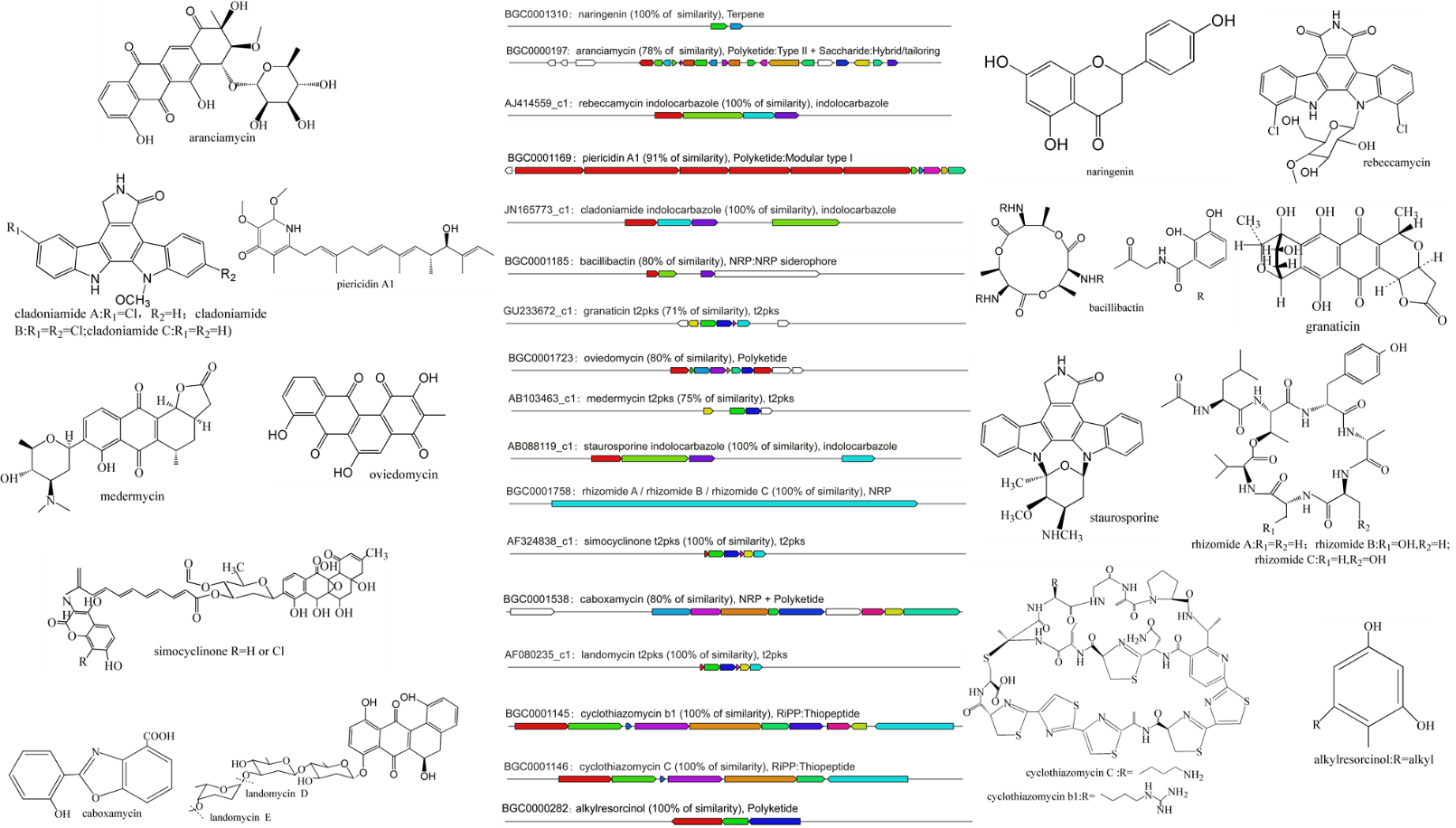


Figure S1

Table S1 Allele sequence accession numbers of the Streptomyces used for the present study

| **Species** | **Strains** | **atpD** | **gyrB** | **recA** | **rpoB** | **trpB** |
| --- | --- | --- | --- | --- | --- | --- |
| *Streptomyces huiliensis* | SCA2-4 ^T^ | JAIQLH000000000 | JAIQLH000000000 | JAIQLH000000000 | JAIQLH000000000 | JAIQLH000000000 |
| *Streptomyces mobaraensis* | NBRC 13819 ^T^ | NZ_AORZ01000143.1 | NZ_AORZ01000007.1 | AORZ01000041.1 | NZ_AORZ01000093.1 | NZ_AORZ01000083.1 |
| *Streptomyces abikoensis* | NBRC 13860 ^T^ | NZ_BMRT01000003.1 | NZ_BMRT01000009.1 | BMRT01000001.1 | NZ_BMRT01000012.1 | NZ_BMRT01000008.1 |
| *Streptomyces orinoci* | NRRL_B-3379 ^T^ | NZ_PHNC01000031.1 | NZ_PHNC01000031.1 | NZ_PHNC01000035.1 | NZ_PHNC01000029.1 | NZ_PHNC01000029.1 |
| *Streptomyces angustmyceticus* | NRRL B-2347 ^T^ | NZ_MUAY01000056.1 | NZ_MUAY01000018.1 | NZ_MUAY01000653.1 | NZ_MUAY01000135.1 | NZ_MUAY01000591.1 |
| *Streptomyces griseocarneus (Streptomyces alboverticillatus)* | NRRL B-24281 ^T^ | NZ_MUFU01000328.1 | NZ_MUFU01000060.1 | NZ_MUFU01000022.1 | NZ_MUFU01000051.1 | NZ_MUFU01000181.1 |
| *Streptomyces catenulae* | NRRL B-2342 ^T^ | NZ_JODY01000004.1 | NZ_JODY01000023.1 | NZ_JODY01000039.1 | NZ_JODY01000013.1 | NZ_JODY01000007.1 |
| *Streptomyces ochraceiscleroticus* | NRRL ISP-5594 ^T^ | NZ_JOAX01000003.1 | NZ_JOAX01000001.1 | NZ_JOAX01000012.1 | NZ_JOAX01000002.1 | NZ_JOAX01000011.1 |
| *Streptomyces varsoviensis* | NRRL ISP-5346 ^T^ | NZ_JOBF01000022.1 | NZ_JOBF01000004.1 | NZ_JOBF01000041.1 | NZ_JOBF01000008.1 | NZ_JOBF01000015.1 |
| *Streptomyces caniferus* | NBRC 15389 ^T^ | NZ_BLIN01000002.1 | NZ_BLIN01000005.1 | NZ_BLIN01000002.1 | NZ_BLIN01000005.1 | NZ_BLIN01000005.1 |
| *Streptomyces sioyaensis* | NRRL B-5408 ^T^ | NZ_JABZEL010000008.1 | NZ_JABZEL010000009.1 | NZ_JABZEL010000008.1 | NZ_JABZEL010000009.1 | NZ_JABZEL010000016.1 |
| *Streptomyces rimosus* subsp*. rimosus* | ATCC 10970 ^T^ | NZ_ANSJ01000029.1 | NZ_ANSJ01000024.1 | NZ_ANSJ01000088.1 | NZ_ANSJ01000031.1 | NZ_ANSJ01000105.1 |
| *Streptomyces libani* subsp.*libani* | NBRC 13452 ^T^ | NZ_BLIP01000001.1 | NZ_BLIP01000001.1 | NZ_BLIP01000001.1 | NZ_BLIP01000001.1 | NZ_BLIP01000001.1 |
| *Streptomyces xinghaiensis* | S187 ^T^ | NZ_CP023202.1 | NZ_CP023202.1 | NZ_CP023202.1 | NZ_CP023202.1 | NZ_CP023202.1 |
| *Streptomyces nigrescens* | AS 4.1410（NBRC 12894）^T^ | FJ406135.1 | FJ406191.1 | FJ406247.1 | FJ406303.1 | FJ406358.1 |
| *Streptomyces pseudoechinosporeus* | NBRC 12518 (NRRL B-16931) ^T^ | KT384693.1 | KT385043.1 | KT385394.1 | KT389014.1 | KT389362.1 |
| *Streptomyces hiroshimensis* | NRRL B-1823（NBRC 3839）^T^ | KT384596.1 | KT384945.1 | KT385294.1 | KT388916.1 | KT389265.1 |

Table S2 MLSA distance values for selected strains in this study

| Strains | MLSA distance (Kimura two-parameter) | | | | | | | | | | | | | | | | |
| --- | --- | --- | --- | --- | --- | --- | --- | --- | --- | --- | --- | --- | --- | --- | --- | --- | --- |
|  | 1 | 2 | 3 | 4 | 5 | 6 | 7 | 8 | 9 | 10 | 11 | 12 | 13 | 14 | 15 | 16 | 17 |
| 1 |  |  |  |  |  |  |  |  |  |  |  |  |  |  |  |  |  |
| 2 | 0.024 |  |  |  |  |  |  |  |  |  |  |  |  |  |  |  |  |
| 3 | 0.066 | 0.065 |  |  |  |  |  |  |  |  |  |  |  |  |  |  |  |
| 4 | 0.619 | 0.625 | 0.607 |  |  |  |  |  |  |  |  |  |  |  |  |  |  |
| 5 | 0.079 | 0.082 | 0.085 | 0.631 |  |  |  |  |  |  |  |  |  |  |  |  |  |
| 6 | 0.068 | 0.073 | 0.041 | 0.597 | 0.079 |  |  |  |  |  |  |  |  |  |  |  |  |
| 7 | 0.081 | 0.086 | 0.076 | 0.630 | 0.054 | 0.078 |  |  |  |  |  |  |  |  |  |  |  |
| 8 | 0.085 | 0.087 | 0.084 | 0.609 | 0.069 | 0.085 | 0.068 |  |  |  |  |  |  |  |  |  |  |
| 9 | 0.076 | 0.081 | 0.079 | 0.601 | 0.082 | 0.071 | 0.080 | 0.081 |  |  |  |  |  |  |  |  |  |
| 10 | 1.020 | 1.019 | 1.011 | 1.092 | 1.042 | 1.003 | 1.024 | 1.015 | 0.991 |  |  |  |  |  |  |  |  |
| 11 | 0.081 | 0.081 | 0.088 | 0.623 | 0.039 | 0.086 | 0.063 | 0.066 | 0.081 | 1.030 |  |  |  |  |  |  |  |
| 12 | 0.082 | 0.088 | 0.082 | 0.606 | 0.073 | 0.083 | 0.071 | 0.061 | 0.077 | 1.026 | 0.070 |  |  |  |  |  |  |
| 13 | 0.089 | 0.089 | 0.089 | 0.635 | 0.040 | 0.087 | 0.064 | 0.073 | 0.088 | 1.055 | 0.035 | 0.070 |  |  |  |  |  |
| 14 | 0.623 | 0.623 | 0.622 | 0.790 | 0.645 | 0.612 | 0.630 | 0.627 | 0.621 | 0.291 | 0.636 | 0.622 | 0.651 |  |  |  |  |
| 15 | 0.608 | 0.613 | 0.626 | 0.140 | 0.626 | 0.620 | 0.645 | 0.626 | 0.604 | 1.137 | 0.620 | 0.631 | 0.625 | 0.809 |  |  |  |
| 16 | 0.617 | 0.620 | 0.632 | 0.131 | 0.609 | 0.625 | 0.642 | 0.643 | 0.625 | 1.132 | 0.611 | 0.630 | 0.587 | 0.798 | 0.117 |  |  |
| 17 | 0.608 | 0.617 | 0.602 | 0.093 | 0.618 | 0.594 | 0.611 | 0.603 | 0.591 | 1.090 | 0.617 | 0.603 | 0.623 | 0.774 | 0.123 | 0.122 |  |

Strains 1.*Streptomyces huiliensis* SCA2-4 ^T^, 2. *Streptomyces mobaraensis* NBRC 13819 ^T^, 3. *Streptomyces abikoensis* NBRC 13860 ^T^, 4. *Streptomyces orinoci* NBRC 13466 ^T^, 5. *Streptomyces angustmyceticus* NRRL B-2347 ^T^, 6. *Streptomyces griseocarneus* (*Streptomyces alboverticillatus*) NRRL B-24281 ^T^, 7. *Streptomyces catenulae* NRRL B-2342 ^T^, 8. *Streptomyces ochraceiscleroticus* NRRL ISP-5594 ^T^, 9. *Streptomyces varsoviensis* NRRL ISP-5346 ^T^, 10. *Streptomyces caniferus* NBRC 15389 ^T^, 11. *Streptomyces sioyaensis* NRRL B-5408 ^T^, 12. *Streptomyces rimosus* subsp.*rimosus* ATCC 10970 ^T^, 13. *Streptomyces libani* subsp.*libani* NBRC 13452 ^T^, 14. *Streptomyces xinghaiensis* S187 ^T^, 15. *Streptomyces pseudoechinosporeus* NBRC 12518 (NRRL B-16931) ^T^, 16. *Streptomyces nigrescens* NBRC AS 4.1410 (12894) ^T^, 17. *Streptomyces hiroshimensis* NRRL B-1823 (NBRC 3839) ^T^

Table S3 Nitrogen-source utilization and antibiotic sensitivity of strain SCA2-4^T^

| **Characteristics** | **Results** |
| --- | --- |
| **Growth on sole nitrogen sources (1.0%, w/v)** |  |
| L-phenylalanine | + |
| Ammonium sulfate | + |
| L-hydroxyproline | + |
| L (+)-cysteine | + |
| Histidine | + |
| Glycine | + |
| Valine | + |
| Ammonium oxalate | + |
| Ammonium acetate | - |
| Ammonium nitrate | - |
| Ammonium molybdate tetrahydrate | - |
| L-arginine | - |
| Glutamate | - |
| **Antibiotic sensitivity (µg/ml)** |  |
| Ampicillin (0.5) | R |
| Chloramphenicol (1.5) | R |
| Streptomycin (0.5) | R |
| Penicillin-G (0.5) | R |
| Gentamicin (1.0) | R |
| Nystatin (5) | R |
| Tetracycline (1.5) | R |
| Neomycin sulfate (0.5) | R |
| Kanamycin sulfate (0.5) | R |
| Rifampicin (0.5) | S |

+: positive reaction; –: negative reaction; S: sensitivity; R: resistance.

Table S4 Functional cluster of orthologous genes (COG) classification of predicted genes in strain SCA2-4^T^

| **COG functional categories** | **Type** | **Gene No.** | % of Gene No. |
| --- | --- | --- | --- |
| **Information storage and processing** |  | **921** | **17.58%** |
| RNA processing and modification | A | 1 |  |
| Chromatin structure and dynamics | B | 1 |  |
| Translation, ribosomal structure and biogenesis | J | 180 |  |
| Transcription | K | 539 |  |
| Replication, recombination and repair | L | 200 |  |
| **Metabolism** |  | **1795** | **34.26%** |
| Energy production and conversion | C | 282 |  |
| Amino acid transport and metabolism | E | 385 |  |
| Nucleotide transport and metabolism | F | 88 |  |
| Carbohydrate transport and metabolism | G | 282 |  |
| Coenzyme transport and metabolism | H | 153 |  |
| Lipid transport and metabolism | I | 181 |  |
| Inorganic ion transport and metabolism | P | 216 |  |
| Secondary metabolites biosynthesis, transport and catabolism | Q | 208 |  |
| **Cellular processes and signaling** |  | **748** | **14.28%** |
| Cell cycle control, cell division, chromosome partitioning | D | 31 |  |
| Cell wall/membrane/envelope biogenesis | M | 179 |  |
| Cell motility | N | 1 |  |
| Posttranslational modification, protein turnover, chaperones | O | 141 |  |
| Signal transduction mechanisms | T | 250 |  |
| Intracellular trafficking, secretion, and vesicular transport | U | 35 |  |
| Defense mechanisms | V | 111 |  |
| **Poorly characterized** |  | **1775** | **33.88%** |
| Function unknown | S | 1775 |  |

| **Cluster ID** | **Type** | **genomic location** | | **Gene No.** | **Most similar known cluster** | **similarity** |
| --- | --- | --- | --- | --- | --- | --- |
|  |  | **Start** | **End** |  |  |  |
| Cluster1 | PKS III-siderophore | 63608 | 104726 | 45 | naringenin biosynthetic gene cluster | 100% |
| Cluster2 | fused | 33740 | 56237 | 22 | ketomemicin B3 / ketomemicin B4 biosynthetic gene cluster | 100% |
| Cluster3 | arylpolyene | 108244 | 130094 | 25 | hygromycin A biosynthetic gene cluster | 31% |
| Cluster4 | terpene | 32261 | 53172 | 22 | 2-methylisoborneol biosynthetic gene cluster | 100% |
| Cluster5 | lantipeptide-lassopeptide | 55186 | 94236 | 39 | moomysin biosynthetic gene cluster | 50% |
| Cluster6 | PKS III | 39841 | 80933 | 44 | - | - |
| Cluster7 | lassopeptide | 1589 | 24191 | 30 | anantin C biosynthetic gene cluster | 75% |
| Cluster8 | terpene | 94506 | 106083 | 10 | - | - |
| Cluster9 | PKS I | 29003 | 73058 | 37 | 5-isoprenylindole-3-carboxylate β-D-glycosyl ester biosynthetic gene cluster | 19% |
| Cluster10 | lantipeptide-NRPS | 1 | 58889 | 47 | SapB biosynthetic gene cluster | 75% |
| Cluster11 | PKS II | 1 | 29654 | 28 | aranciamycin biosynthetic gene cluster | 78% |
| Cluster12 | other | 65173 | 88774 | 27 | salinamide A biosynthetic gene cluster | 21% |
| Cluster13 | ladderane | 1194 | 43602 | 36 | 5-isoprenylindole-3-carboxylate β-D-glycosyl ester biosynthetic gene cluster | 14% |
| Cluster14 | PKS I | 29840 | 73751 | 42 | meilingmycin biosynthetic gene cluster | 3% |
| Cluster15 | PKS I | 45204 | 83115 | 35 | - | - |
| Cluster16 | other | 1 | 31807 | 23 | - | - |
| Cluster17 | indole | 48576 | 72322 | 19 | AT2433/Rebeccamycin/Staurosporine/K-252a/Cladoniamide biosynthetic gene cluster | >75% |
| Cluster18 | siderophore | 3215 | 14909 | 11 | kinamycin biosynthetic gene cluster | 20% |
| Cluster19 | PKS I | 1 | 32504 | 32 | hygromycin A biosynthetic gene cluster | 6% |
| Cluster20 | PKS I | 1 | 60356 | 48 | A-503083 A / A-503083 B / A-503083 E / A-503083 F biosynthetic gene cluster | 7% |
| Cluster21 | PKS III | 1 | 35286 | 29 | violapyrone B biosynthetic gene cluster | 28% |
| Cluster22 | PKS III-terpene | 8574 | 64323 | 48 | flaviolin biosynthetic gene cluster | 50% |
| Cluster23 | terpene | 8554 | 29790 | 19 | - | - |
| Cluster24 | NRPS | 1 | 31692 | 27 | bacillibactin biosynthetic gene cluster | 80% |
| Cluster25 | lassopeptide | 1 | 46295 | 54 | lagmysin biosynthetic gene cluster | 80% |
| Cluster26 | terpene-NRPS | 1 | 56046 | 40 | geosmin biosynthetic gene cluster | 100% |
| Cluster27 | terpene | 2849 | 23769 | 18 | - | - |
| Cluster28 | terpene | 1 | 38268 | 42 | curacomycin biosynthetic gene cluster | 15% |
| Cluster29 | PKS III | 10816 | 45677 | 29 | flaviolin biosynthetic gene cluster | 50% |
| Cluster30 | NRPS | 1560 | 44843 | 40 | arylomycin biosynthetic gene cluster | 22% |
| Cluster31 | bacteriocin | 14255 | 25119 | 14 | - | - |
| Cluster32 | PKS I | 1 | 29812 | 19 | piericidin A1 biosynthetic gene cluster | 91% |
| Cluster33 | otherks | 11880 | 39620 | 11 | - | - |
| Cluster34 | PKS I -NRPS | 1 | 38417 | 17 | microsclerodermin biosynthetic gene cluster | 21% |
| Cluster35 | PKS II | 1 | 32445 | 33 | Oviedomycin/Landomycin/Simocyclinone/Granaticin/Medermycin biosynthetic gene cluster | >71% |
| Cluster36 | siderophore | 17638 | 29297 | 12 | - | - |
| Cluster37 | terpene | 14701 | 33076 | 25 | - | - |
| Cluster38 | NRPS | 1 | 29103 | 23 | rhizomide A / rhizomide B / rhizomide C biosynthetic gene cluster | 100% |
| Cluster39 | lantipeptide | 1 | 14141 | 18 | - | - |
| Cluster40 | PKS I | 1 | 24417 | 20 | A54145 biosynthetic gene cluster | 6% |
| Cluster41 | lantipeptide | 1 | 21354 | 16 | chejuenolide A / chejuenolide B biosynthetic gene cluster | 7% |
| Cluster42 | PKS I | 1 | 19986 | 7 | piericidin A1 biosynthetic gene cluster | 50% |
| Cluster43 | terpene | 945 | 19474 | 19 | hopene biosynthetic gene cluster | 53% |
| Cluster44 | PKS I | 1 | 19130 | 10 | - | - |
| Cluster45 | NRPS | 1 | 16852 | 15 | matlystatin A biosynthetic gene cluster | 38% |
| Cluster46 | thiopeptide | 1 | 16821 | 12 | cyclothiazomycin b1/ cyclothiazomycin c biosynthetic gene cluster | 100% |
| Cluster47 | otherks | 1 | 16490 | 12 | caboxamycin biosynthetic gene cluster | 80% |
| Cluster48 | NRPS | 1 | 14825 | 2 | cysteoamide biosynthetic gene cluster | 18% |
| Cluster49 | PKS III | 1 | 9899 | 8 | alkylresorcinol biosynthetic gene cluster | 100% |
| Cluster50 | other | 1 | 9715 | 9 | cadaside A / cadaside B biosynthetic gene cluster | 19% |
| Cluster51 | terpene | 1 | 7754 | 6 | - | - |

Table S5 Biosynthetic gene clusters of secondary metabolites of strain SCA2-4*^T^* on the online antiSMASH v 6.0.1

Table S6 Cluster number and gene number shown in different cluster types of strain SCA2-4^T^

| **Cluster Type** | **Cluster Number** | **Gene Number** |
| --- | --- | --- |
| PKS I | 9 | 250 |
| terpene | 8 | 161 |
| NRPS | 5 | 107 |
| PKS III | 4 | 110 |
| other | 3 | 59 |
| lassopeptide | 2 | 84 |
| PKS II | 2 | 61 |
| siderophore | 2 | 23 |
| otherks | 2 | 23 |
| lantipeptide | 2 | 34 |
| PKS III-siderophore | 1 | 45 |
| fused | 1 | 22 |
| arylpolyene | 1 | 25 |
| lantipeptide-lassopeptide | 1 | 39 |
| lantipeptide-NRPS | 1 | 47 |
| ladderane | 1 | 36 |
| indole | 1 | 19 |
| PKS III-terpene | 1 | 48 |
| terpene-NRPS | 1 | 40 |
| bacteriocin | 1 | 14 |
| PKS I-NRPS | 1 | 17 |
| thiopeptide | 1 | 12 |
| **total** | **51** | **1276** |

Table S7 Inhibitory activity and MIC of extracts of strain SCA2-4 ^T^ against Foc TR4

| **Index** | **results** |
| --- | --- |
| Inhibition zone (mm) | 31.83±2.36 |
| Mycelial inhibition (%) | 42.47 |
| MIC of extracts (µg/ml) | >6.25 |
| MIC of cycloheximide (µg/ml) | >1.563 |
| MIC of nystatin (µg/ml) | >6.25 |
